# The association between blood pressure level and serum uric acid concentration in hemodialysis patients

**DOI:** 10.12860/jnp.2015.16

**Published:** 2015-07-01

**Authors:** Jamshid Roozbeh, Mohammad-Mahdi Sagheb, Elaheh Vafaie

**Affiliations:** ^1^Shiraz Nephro-Urology Research Center, Shiraz University of Medical Sciences, Shiraz, Iran; ^2^Department of Nephrology, Shiraz University of Medical Sciences, Shiraz, Iran; ^3^Department of Internal Medicine, Namazee Hospital, Shiraz University of Medical Sciences, Shiraz, Iran

**Keywords:** Uric acid, Blood pressure, Hemodialysis

## Abstract

*Background:* High blood pressure is a common condition in hemodialysis patients. Uric acid, which is high in these patients due to decreased clearance, had been shown to positively correlate with blood pressure in animal studies.

*Objectives:* The goal of this investigation was to evaluate the impact of high uric acid level on blood pressure in these patients.

*Patients and Methods:* Ninety-one patients, on three times weekly hemodialysis, were studied. Uric acid levels were measured just before and after hemodialysis along with blood pressures before, during and after each session. Data were analyzed by SPSS 15. A P value less than 0.05 was considered significant.

*Results:* 40 (44%) of patients had serum uric acid ≥6 mg/dl. Before dialysis 51 (61%) and 19 (21%) had high systolic blood and diastolic blood pressures respectively. Also, 50 (55%) were with wide pulse pressure and 63 (69%) had high mean arterial pressure (MAP). Additionally 62 (68%) developed inter-dialysis hypotension. After measuring odds ratio for hyperuricemia in each group, we observed low risk of hypruricemia in the group with high systolic pressure (OR = 0.352; 95% CI: 0.147-0.844; *P* = 0.01), the high MAP group (OR = 0.382; 95% CI: 0.153-0.955; *P* = 0.03) and wide pulse pressure group (OR = 0.416; 95% CI: 0.177-0.975; *P* = 0.04). There was no association between high uric acid level and diastolic pressure (*P* = 0.11) and inter-dialysis hypotension (*P* = 0.33). No relationship was found between serum uric acid and KT/V (*P* = 0.2), normalized protein catabolic rate (nPCR) (*P* = 0.07) and body mass index (BMI) (*P* = 0.4).

*Conclusions:* This study showed paradoxical association between high uric acid level and high systolic pressure, high MAP and wide pulse pressure and these effects were independent of dialysis duration, dialysis efficacy and nutrition, assuming that these relationships could be due to reverse epidemiology in dialysis patients.

**Implication for health policy/practice/research/medical education:**

Blood pressure variation is very important in hemodialysis patients. As uric acid is a proven factor in blood pressure in animals and humans, finding its association with hypertension in hemodialysis patients can be a promising therapeutic gold.

## 1. Background

Numerous studies during last decades showed strong relationship between uric acid and hypertension (1-4). Uric acid acts as antioxidant in endothelial cells against inflammatory cells and also maintains vascular dilatation during oxidative stress (5-10).But in high levels it can cause hypertension, diabetes mellitus, metabolic syndrome and cardiovascular diseases (11-13). The pathogenesis of uric acid induced hypertension had been studied in rats and humans and includes endothelial dysfunction, decreased levels of serum nitrites, increased renin and decreased nitric oxide synthase expression in kidney and renal arteriolar vasoconstriction (14-16).

Kidneys excrete approximately 60% to 70% of total uric acid from body (17). Uric acid is a small molecule that can be partially removed by hemodialysis as for urea (18). Paradoxical results were gained between hyperuricemia and outcomes in chronic kidney disease (CKD) patients. In one study, increased mortality was seen in hemodialysis patients that had the highest and the lowest quintiles of uric acid (19). Another study showed increased mortality in stage 3-4 of CKD due to all-cause mortality and cardiovascular disease in hyperuricemic patients (20).

Hypertension is a common finding in hemodialysis patients, occurring in 75% of them and it is a significant factor in cardiovascular disease, stroke and renal failure (21,22). Wide pulse pressure and interdialysis hypotension are also associated with increased long-term mortality in dialysis patients (23). The role of uric acid in hypertension in end-stage renal disease (ESRD) was studied once in which Silverstein and colleague (24) showed a positive relationship between high uric acid and hypertension in pediatric hemodialysis patients.

## 2. Objectives

The aim of this study was to investigate the relationship between serum uric acid levels and blood pressure both as modifiable risk factors in morbidity and mortality in adult hemodialysis patients.

## 3. Patients and Methods

### 3.1. Study & Population

Patients (19 to 79 years old) including in this cross-sectional study were males and females above 18 years old on 4 hours hemodialysis, 3 times per week for at least 3 consecutive months in 3 hemodialysis centers in Shiraz, Iran. No drugs that could alter blood pressure were administered during dialysis. Patients receiving medications with impact on uric acid level (allopurinol and diuretic) were excluded. One patient had in remission malignancy (multiple myeloma) and one patient had inactive rheumatologic disease (Sjogren syndrome). No one had liver or infectious disease.

### 3.2. Blood pressure

Blood pressures were measured by 1 automatic sphygmomanometer in hemodialysis center by a nurse just before and after dialysis and every 1 hour during dialysis in 3 consecutive sessions. According to KDOQI guideline, hypertension was defined as systolic pressure ≥140 mm Hg and diastolic pressure ≥90 mm Hg, high mean arterial pressure (MAP) as pressure ≥97 mm Hg, interdialysis hypotension as a decrease of SBP>20 mm Hg from basal value associated with signs and symptoms. Also wide pulse pressure was recorded as ≥60 mm Hg difference between systolic and diastolic pressure before dialysis.

### 3.3. Weight measurement and efficacy of hemodialysis

Weights were measured before and after dialysis and KT/V was calculated for each patient.

### 3.4. Laboratory methods

Blood samples were taken from patients just before dialysis from arterial line for blood urea nitrogen (BUN), uric acid, albumin before dialysis. BUN and uric acid after dialysis were measured too.

### 3.5. Nutrition

As nutrition can effect level of uric acid, nutrition status was assessed by normalized protein catabolic rate (nPCR). Generally the nPCR shows the amount of protein taken by patient and uric acid is a product of protein. Also body mass index (BMI) was calculated for each patient.

### 3.6 Antihypertensive medication

Of all patients, 52% were took antihypertensive medication (except for diuretics).

### 3.7. Ethical issues

1) The research followed the tenets of the Declaration of Helsinki; 2) informed consents was obtained; and 3) the research was approved by ethical committee of Shiraz University of Medical Science.

### 3.8. Statistical analysis

All data were analyzed by SPSS 15 (SPSS Inc., Chicago, IL, USA). Values were shown as the mean±standard deviation (SD) or as percent frequency. Correlation analysis between 2 values was performed by Pearson correlation test. Association between uric acid and blood pressures was calculated by odds ratio using multiple regression analysis. A *P* value <0.05 was considered significant.

## 4. Results

### 4.1. Demographic data

This study included 60 (65.9%) males and 31 (34.1%) females. The average age was 50.8±16.7 years. Data are shown in Table 1.

**Table 1:** Baseline characteristics of patients

**Characteristics**	**Value**
Age (y)	50.8±16.7
Dialysis vintage (mon)	35.6±26.8
KT/V	1.1±0.36
BMI (kg/m²)	24.1±5.4
nPCR (g/kg/d)	0.87±0.27
Albumin (g/dl)	3.9±0.36
Predialysis systolic pressure (mm Hg)	144.1±22.9
Predialysis diastolic pressure (mm Hg)	81.8±12.04
Predialysis MAP (mm Hg)	102±14.4
Predialysis uric acid (mg/dl)	5.7±1.02

### 4.2. Serum uric acid

The average predialysis and postdialysis uric acid level was 5.7±1.02 mg/dl and 3.2±0.99 mg/dl, respectively. In this study, 40.91 (44%) had predialysis serum uric acid ≥6 mg/dl. Only 1 patient had serum uric acid ≥6 mg/dl after dialysis; thus association between postdialysis uric acid and other variables was not possible.

### 4.3. Dialysis efficacy

There was no relationship between uric acid and Kt/V (*P *= 0.212).

### 4.4. Weight

No association was found between uric acid and BMI (*P *= 0.402).

### 4.5. Nutritional status

No relationship was observed between uric acid and nPCR (*P *= 0.071).

### 4.6. Dialysis vintage

There was no relationship between uric acid and duration of dialysis (*P *= 0.273).

### 4.7. Blood pressure

Of all patients, 56 (61.5%) had high systolic blood pressure. Also 19 (20.9%) had high diastolic pressure. Likewise 63 (69.2%) had high MAP, and 50 (54.9%) had high wide pulse pressure too. Additionally 62 (68.1%) developed interdialysis hypotension.

### 4.8. Association between uric acid and blood pressures

Odds ratio for high uric acid level in the group with predialysis systolic hypertension was 0.352 (95% CI: 0.147- 0.844; *P *= 0.01); for high MAP was 0.382 (95% CI: 0.153-0.955; *P *= 0.03); for wide pulse pressure was 0.416 (95% CI: 0.177-0.975; *P *= 0.04); for predialysis diastolic hypertension was 0.407 (95% CI: 0.0.131-1.25; *P *= 0.11); and for interdialysis hypotension was 0.648 (95% CI: 0.266-1.577; *P *= 0.33). Therefore, there is lower risk for systolic hypertension, high MAP and wide pulse pressure in the group with high uric acid level Associations are summarized in Table 2 and 3.

**Table 2:** Blood pressure according to uric acid level

**Blood pressure**	**Uric acid ≥6 mg/dl**	**Uric acid <6 mg/dl **
Predialysis systolic hypertension	19 (34.5%)	36 (65.5%)
Predialysis diastolic hypertension	5 (27.8%)	13 (72.2%)
Predialysis high MAP	23 (37.1%)	39 (62.9%)
Wide pulse pressure	17 (34.7%)	32 (65.3%)
Interdialysis hypotension	25 (41%)	36 (59%)

**Table 3:** Odds ratio for high uric acid level

**Blood pressure**	**Odds ratio**	***P*** ** value**
Predialysis systolic hypertension	0.352	0.01
Predialysis diastolic hypertension	0.407	0.1
Predialysis high MAP	0.382	0.03
Wide pulse pressure	0.416	0.04
Interdialysis hypotension	0.648	0.3

## 5. Discussion

Hypertension is a common finding in hemodialysis patients and it occurs in up to 85% of them (21). In these patients multiple factors can cause high blood pressure including fluid overload, activation of rennin angiotensin-aldosterone axis or use of exogenous erythropoietin that make it difficult to evaluate the causative mechanism (25,26).

Uric acid levels are high in renal failure patients due to decreased clearance (17). Dialysis can remove uric acid from blood partially (18). Higher uric acid levels are shown to be associated with higher mortality in hemodialysis patients (20).

In this study we tried to elucidate the relationship between uric acid and blood pressure. We observed low frequency of high uric acid levels in our patients. Occurrence of uric acid ≥6 mg/dl before treatment was 44% (40.91) and only 1 patient had this level after dialysis. Another important observation was paradoxical relationship between high uric acid and high systolic blood pressure, high MAP and wide pulse pressure. These associations were independent of dialysis duration. Dialysis efficacy, BMI and albumin confirmed that nutrition and protein breakdown did not affect uric acid level.

These findings were contrary to previous studies in literature in animals and humans. The most significant of these studies were 3 trials that induced hyperuricemia in rats can cause hypertension after 3 weeks in mazzali experiment. Mechanism of hypertension in these rats were included renal artery vasoconstriction, arteriolopathy, decreased nitric oxide and increased oxidants and angiotensin in kidney. Lowering uric acid with drugs like allopurinol or using radical scavengers, to reverse intrarenal oxidative stress induced by uric acid,blood pressures were returned to normal. (15,27,28). In human studies, Park et al (29) showed that high uric acid was associated with arterial stiffness in postmenopausal women. Arterial stiffness can cause interdialysis hypertension in dialysis patients (30). Another longitudinal study in 9104 humans with normal renal function with different race and sex showed increased risk of hypertension in hyperuricemic group (1). In dialysis patients this association was once conducted in 46 hemodialysis children in which predialysis systolic pressure was positively related to high uric acid level which was independent of volume, weight and nutrition (23).

Despite these observations, lowering uric acid level was not effective in lowering mortality and CKD progression (31), to the extent, that high uric acid had shown to have protective effect on mortality of hemodialysis patients, while it is a marker of better nutrition in this group and also acts as antioxidant in cardiovascular system (32).

The present study can be another example of reverse epidemiology as described previously that high BMI is a survival factor in maintenance hemodialysis patients or by Liu et al that high cholesterol level is inversely related to dialysis mortality (33,34). The “reverse epidemiology” (35) or “dialysis-risk-paradox” (36,37) phenomena were concluded from several epidemiologic studies that found reverse relationship between mortality in hemodialysis patients and obesity (38), blood pressure (39), creatinine (40), cholesterol (36), and homocysteine (41). Is this hypothesis really exist or not and its impact on mortality is doubtful.

## 6. Conclusions

We showed that high uric acid levels can be a marker of protection against high systolic blood pressure, high MAP and wide pulse pressure, all of which can increase mortality in hemodialysis patients.

## 7. Limitations of the study

Our study has some pitfalls. Firstly, we did not investigate the effect of lowering uric acid with drug on blood pressure. Secondly, larger group could be better for conducting this study and also patients under peritoneal dialysis can be included. In fact, we could only consider 91 patients which most of them had low uric acid level. Larger group in a longer period of time may be needed to study this aspect of dialysis. Additionally, we did not find any association between uric acid and markers of nutrition as they affect uric acid level and we also did not consider the effect of residual renal function on uric acid and blood pressure.

## Acknowledgments

This work was extracted from thesis of Elaheh Vafaie at the internal medicine department of Shiraz University of Medical Sciences (Registered code; 3037).

## Authors’ contribution

Collecting data: EV. Statistical analysis: MMS and EV. Drafting manuscript: EV and MMS. Study supervision: JR and MS.

## Conflicts of interest

The authors declared no competing interests.

## Funding/Support

The Research Council of Shiraz University of Medical Sciences financially supported this project (grant No. 90-01-01-3037).
